# Treatment of horizontal root fracture: a case report

**DOI:** 10.4076/1757-1626-2-8101

**Published:** 2009-06-19

**Authors:** Stefania Cantore, Andrea Ballini, Vito Crincoli, Felice Roberto Grassi

**Affiliations:** Department of Dental Sciences and Surgery, University of BariP.zza Giulio Cesare n.11, Bari, 70124Italy

## Abstract

Radicular fractures in permanent teeth are uncommon injuries among dental traumas, being only 0.5-7% of the cases. Traumatic dental injuries occur more frequently in young patients, and vary in severity from enamel fractures to avulsions.

The magnitude of these problems is confirmed by statistical data on the prevalence of dental trauma during childhood and adolescence.

Fracture occurs often in the middle-third of the root and rarely at the apical-third. The present paper reports a clinical case of a horizontal radicular fracture located between the middle- and apical-third of a upper left-central incisors followed-up over 4 years.

## Case presentation

In this work a case report of a 14 years old Caucasian boy has been described. The patient had a scooter crash without any protective head and body gear [1] and referred to our attention only 4 hours after the accident.

**Figure 1.**
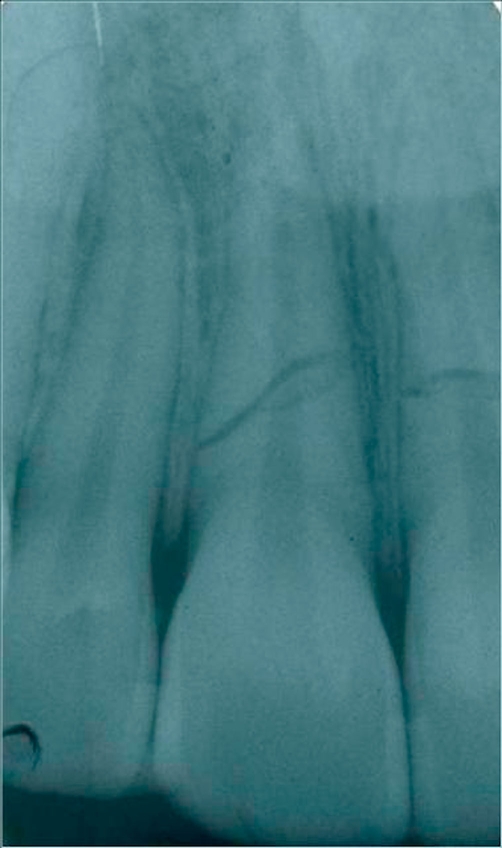
A horizontal root fracture was presented radiographically localized between the third middle and the third apex of the central superior incisors. The fragments appeared to be separated by a radiolucent line, and the fractured edges were rounded.

As a consequence he underwent a dentoalveolar damageof both upper central incisors and vestibolarization of two crown fragments, with no fracture line clinically detectable. A horizontal root fracture was radiographically evident between the middle third and the apical third of the central upper incisors (Figure 1).

**Figure 2.**
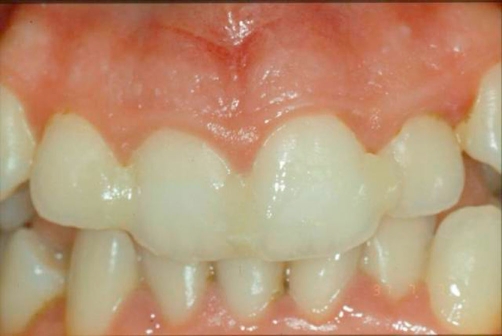
Reduction of crown fragments and splinted all the frontal elements with interproximal composite.

**Figure 3.**
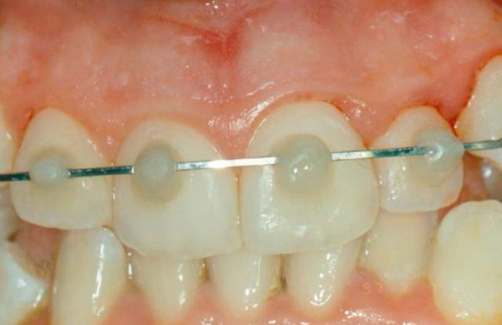
Orthodontic hard immobilization with a floss in stainless steel fixed diameter with photopolymeric resin.

The care plan comprised reduction, repositioning and rigid splinting of the coronal fragments. [2]. The initial treatment consisted in repositioning, using firm finger pressure to the coronal segments. All the maxillary elements were subsequently splinted using interproximal composite (Figure 2). After 24 hours an orthodontic stainless steel arch was applied, using a photopolymeric resin after a careful assessment of the occlusal contacts (Figure 3). No medication was prescribed. The splint was held 1 year long because of the presence of severe dental mobility. Despite this therapeutic solution, a correct oral hygiene was kept thanks to a professional hygiene, to a strong motivation of the patient and to the use of the dental floss (Superfloss^®^). Moreover, the absence of the fracture line with the oral environment prevented any bacterial penetration. Ten days later the damaged teeth did not show chromatic alterations and the thermal and electrical tests (Crio Test - Pulp Test) suggested no pulpar necrosis [3].

**Figure 4.**
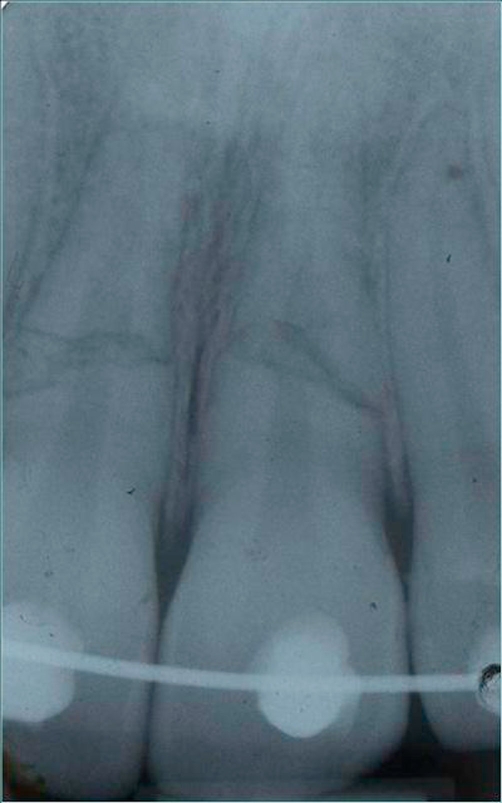
RX after orthodontic hard immobilization with a floss in stainless steel.

**Figure 5.**
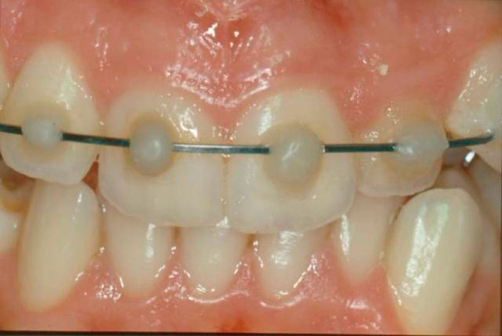
Clinical control at 3 months.

**Figure 6.**
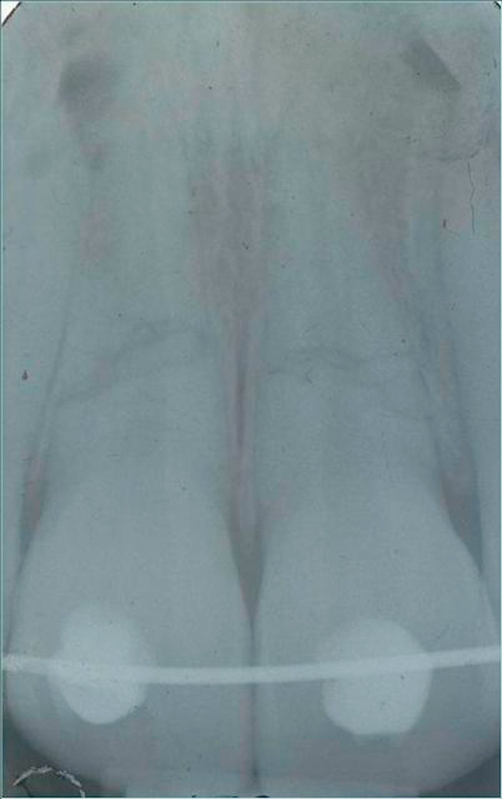
Radiographic control at 3 months.

**Figures 7-9.**
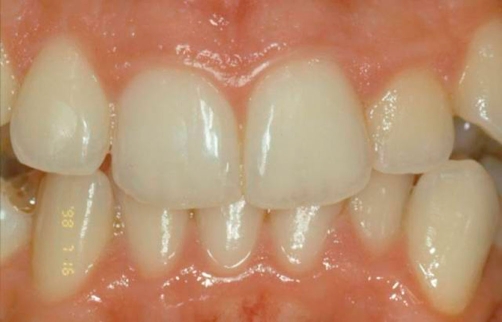

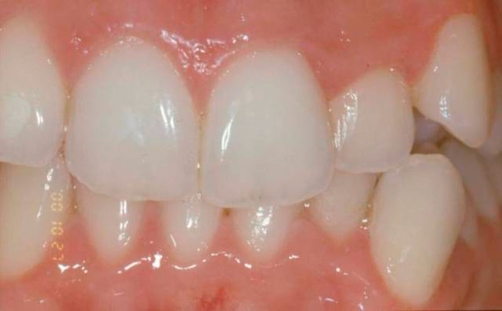

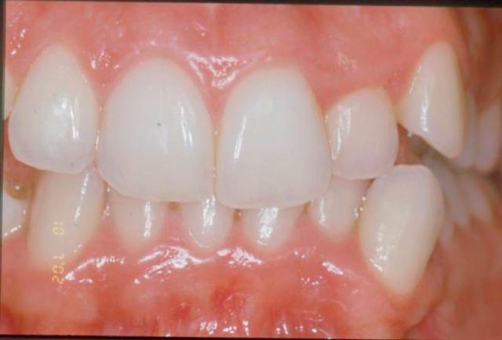
Clinical control at 1, 3 and 4 years.

**Figure 10.**
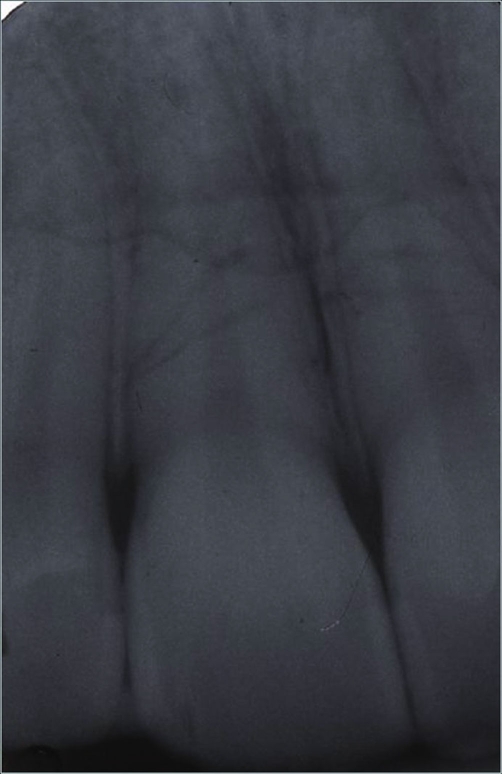
Radiographic control at 4 years.

A follow-up was performed after 20 and 30 days, through clinical and radiographic examinations. Then the patient returned for periodic clinical and radiographic follow-up after 3 months and 1, 3 and 4 years (Figures 4-10). After the splint removal the mobility of both incisors was within normal limits and the patient reported no discomfort with his teeth and no pain during horizontal and vertical percussion tests. The electrical test responses of both central incisors were grade 4. The control check was performed on laterals and it was grade 2. No sign of pathology was visible on the radiograms.

## Discussion and conclusions

The prognosis of root fractures depends on the extent of the fracture line, the pulp tissue situation, occlusion, dislocation of fragments and the general health of the patient [1]. According to Andreasen and Hjørting-Hansen [4], there are 4 healing patterns, and preinjury and injury factors can affect the prognosis and tissue response to dental trauma [5].

1. Healing with tissue, giving union across the fracture.
2. Healing with interposition of hard and soft tissue between the fragments.
3. Healing with interposition of only soft tissue.
4. No healing.

The International Association of Dental Traumatology has recently developed a consensus statement on diagnosis and treatment of dental traumas [6]. According to these guidelines, a correct care plan should be performed through clinical and radiographic examinations, followed by sensibility tests and patient care instructions.

If the fracture line is in communication with the oral cavity, the immobilization is difficult and microbial contamination of the pulp with subsequent pulpal necrosis is almost inevitable [3]. Dental pulp necrosis may be reported from 20 to 44% of the root fracture cases whereas in luxated teeth without fracture, necrosis occurs in at least 43.5% of cases [5,7-9].

Successful management of root fractures often involves a multidisciplinary combination of endodontic, orthodontic, periodontic and prosthetic therapy [1,3].

Treatment options with root fractures typically include reduction of the fracture and stabilization by rigid fixation for a variable time [5]. According to Andreasen, splinting may be applied within a week [3].

Nowadays, splinting for 1-3 months is recommended, but no study on the effects of the splinting period on prognosis has been carried out yet [5,6,10]. In our case, both maxillary central incisors had severe mobility and dislocation, therefore a prolonged duration of the fixed appliance was considered safer and viable for healing.

Many investigators have suggested that the reversal of vitality of root-fractured teeth vary between a few months and 2 years [3,11-13]. In case of horizontal root fractures successful results have been reported, with success rates ranging from 54% to 77% of cases [5].

In a recent study Andreasen investigated the healing of 400 root fractures, and the results showed that the type of splints appeared to have no association with the healing outcome [14] and has also stated that the location of the root fracture does not affect pulp survival [1,3,5,14].

Root canal therapy is indicated when vitality control reveals non-vital pulp tissue, or if the patient complains of pain or discomfort of the tooth. [7,11,13]. Repair appears to depend on an intact periodontal ligament, from which the hard tissue forming cells originate [4]. However, healing of root fractures without treatment is also presented in many reports [12,13,15]. In traumatic injuries, follow-up is of critical importance [16]. As illustrated in our cases, after 4 years fragments steadily healed and pulp is still vital without complications by using orthodontic wire. In this way we can prevent further occlusal trauma that could negatively influence the survival of the teeth [2].

We can conclude that the primary purpose of the treatment of fractured elements is to keep a steady tooth and, when it's possible, its vitality. It is important to remember that the maintenance of a natural tooth during growth could be an excellent intermediate solution before implant rehabilitation [1,17].

## Abbreviations

None.
